# *Providencia pseudovermicola* sp. nov.: redefining *Providencia vermicola* and unveiling multidrug-resistant strains from diabetic foot ulcers in Egypt

**DOI:** 10.1186/s12866-025-03927-3

**Published:** 2025-04-23

**Authors:** Samira M. Hamed, Manal M. Darwish, Reham Monir, Ahmed Al Taweel, Ayat I. Ghanem, Ihab N. Hanna, Mai A. Amer

**Affiliations:** 1 Microbiology and Immunology Department, Faculty of Pharmacy, October University for Modern Sciences and Arts (MSA), Giza, Egypt; 2 Medical Microbiology and Immunology, Faculty of Medicine, Ain Shams University, Cairo, Egypt; 3 National Institute of Diabetes and Endocrinology (NIDE), Cairo, Egypt

**Keywords:** Diabetic foot ulcer, *Providencia* spp., *Providencia vermicola*, *Providencia pseudovermicola*, Whole genome sequencing, Taxonogenomics, OGRIs, ANI, dDDH, Multidrug resistance

## Abstract

**Background:**

*Providencia* species are concerning due to their intrinsic resistance to colistin and tigecycline, complicating the treatment of multidrug-resistant (MDR) infections.

**Methods:**

In the current study, two MDR isolates, DFU6 and DFU52^T^, were recovered from infected diabetic foot ulcers in Egypt in 2024. Following their initial identification as *Providencia stuartii* using VITEK® 2 and MALDI-TOF-MS, the isolates were subjected to whole-genome sequencing via DNBseq.

**Results:**

While the 16S rRNA gene showed 100% similarity to that of *Providencia vermicola*, phylogenomic analysis against the type strains in the TYGS database, including *P. vermicola* DSM 17385^T^ confirmed that these isolates represent a distinct species within the genus, further supported by overall genome-relatedness indices (ORGIs). This discrepancy prompted us to revise the taxonomy of all published genomes of *P. vermicola* strains (*n* = 59) which revealed misidentification of at least 56 strains that are unrelated to the type strain of this species. DFU6 and DFU52^T^ carried novel sequence types (ST29 and ST41, submitted to PubMLST) and harbored multiple resistance genes. Both strains contained the *qnrD1* gene on a small, non-mobilizable plasmid. DFU52^T^ possessed a conjugative plasmid encoding *bla*_CMY−6_, *bla*_NDM−1_, *rmtC*, *aac(6’)-Ib10*, *sul1*, *aph(3’)-Ia*, and *qacEΔ1*. DFU6 carried an IS*Ecp*1-associated *bla*_CTX−M−14_, along with *aadA*, *dfrA1*, *lnuF* in a class 2 integron, and *armA*, *msrE*, and *mphE* on a resistance plasmid. Both isolates also featured a pathogenicity island (PAI) integrated into the *pheV* gene with fimbriae-encoding genes.

**Conclusion:**

Following our reassessment of the taxonomic classification of all *P. vermicola* strains with published genomes, we propose reclassifying certain strains, including DFU6 and DFU52^T^, into distinct species for which we propose the name *Providencia pseudovermicola* sp. nov. We recommend DFU52^T^ (= CCASU-2024-72) as the type strain for the novel species. We also shed light on the public health threat of this novel species as a human pathogen that harbours carbapenem and aminoglycoside resistance genes on mobile genetic elements.

**Supplementary Information:**

The online version contains supplementary material available at 10.1186/s12866-025-03927-3.

## Introduction

*Providencia* spp. are non-lactose fermenting, Gram-negative rods belonging to the family *Morganellaceae* within the order *Enterobacterales* [1]. Members of the genus *Providencia* can thrive in diverse environments [2] and have been isolated from various organisms [3].

*Providencia* is a rapidly evolving genus, with at least 18 species identified to date as per the List of Prokaryotic names with Standing in Nomenclature (LPSN), available at: https://lpsn.dsmz.de/genus/providencia. Although they are primarily opportunistic pathogens that predominantly affect immunocompromised and hospitalized patients [4], *Proviencia* spp. pose a significant public health concern. This is due to their ability to cause severe infections in vulnerable patients, combined with their intrinsic resistance to several last-resort antibiotics, such as colistin and tigecycline [5]. Moreover, growing evidence points to the emergence of extended-spectrum ß-lactamase (ESBL)- and carbapenemase-producer strains, further limiting treatment options [6–11]. The genus *Providencia* has been associated with various types of infections, most commonly urinary tract infections, gastroenteritis, bloodstream infections, and wound infections [12]. *Providencia* infections have been associated with high mortality rates [13]. Among all known *Providencia* species, *Providencia rettgeri*, *Providencia stuartii*, and *Providencia alcalifaciens* are the most common clinical MDR species and are known to be the primary cause of several nosocomial outbreaks [7, 12, 14, 15].

Remarkably, six new *Providencia* species have been discovered in the past five years, driven by advancements in whole genome sequencing (WGS) [16–20]. However, these developments have also raised concerns about the taxonomy of some *Providencia* species and the validity of species assignments for certain type strains. Dong, et al. [20] have recently redefined the species *Providencia thailandensis* as *P. stuartii* based on their average nucleotide identity (ANI) and digital DNA-DNA hybridization (dDDH) values. A few years earlier, Andolfo, et al. [21] published the genome sequence of the *Providencia vermicola* type strain DSM 17385^T^. Using multilocus sequence typing with different marker sets, they revealed that none of the previously published *P. vermicola* genomes accurately represent this taxonomic species. The authors hence called for a reevaluation of the taxonomy of some *P. vermicola* strains with published genomes.

As the first report in Egypt, we present the genomes of two multidrug-resistant (MDR) *Providencia* sp. isolates recovered from infected diabetic foot ulcers (DFUs) in diabetic patients in Egypt. Genome-based phylogenetic analysis revealed that these isolates cluster with *P. vermicola* genomes previously reported by Andolfo, et al. [21] to be unrelated to the type strain of this species. After reassessing the taxonomic classification of all *P. vermicola* strains with published genomes, we propose the reclassification of certain *P. vermicola* strains, including ours, into a distinct species. We suggest the name *Providencia pseudovermicola* sp. nov. to reflect the previous misclassification and present one of our strains, DFU52^T^ (= CCASU-2024-72), as the type strain for this novel *Providencia* species.

## Materials and methods

### Collection and preliminary identification of bacterial isolates

DFU6 and DFU52^T^ were obtained from the microbiology laboratory of the National Institute of Diabetes and Endocrinology (NIDE). DFU6 was collected in February 2024 from a 52-year-old female patient, and DFU52^T^ was obtained in March 2024 from a 43-year-old female patient. The isolates were received by the microbiology laboratory for microbiological identification and antimicrobial susceptibility testing as part of the routine clinical care of the patients admitted to the institute with infected DFUs. After identification using conventional microbiological techniques, the isolates were identified by Matrix-assisted laser desorption/ionization time-of-flight mass spectrometry (MALDI-TOF-MS) (BioMérieux; Marcy l’Etoile, France) and the VITEK® 2 automated system (bioMérieux, Lyon, France), using Gram-negative identification cards (GN ID card).

### Antimicrobial susceptibility testing

The susceptibility of the two strains to a large panel of antimicrobial agents was evaluated using the Kirby-Bauer disc diffusion method. The following antimicrobial discs (Oxoid, UK) were utilized: amoxicillin/clavulanic acid (20/10 μg), piperacillin/tazobactam (100/10 μg), cefoxitin (30 μg), ceftriaxone (30 μg), cefotaxime (30 μg), cefepime (30 μg), aztreonam (30 μg), meropenem (10 μg), gentamicin (10 μg), amikacin (30 μg), ciprofloxacin (5 μg), tetracycline (30 μg), tigecycline (15 μg), chloramphenicol (30 μg), nitrofurantoin (300 μg), fosfomycin (200 μg), and trimethoprim/sulfamethoxazole (1.25/23.75 μg). Additionally, the broth microdilution assay was employed to determine the minimum inhibitory concentrations (MICs) of colistin (Sigma-Aldrich, St Louis, MO, USA) across a concentration range of 128–0.125 μg/ml. All susceptibility tests were conducted and interpreted according to the Clinical and Laboratory Standards Institute (CLSI) guidelines [22] for all antimicrobial agents, except for tigecycline, for which the susceptibility breakpoints recommended by EUCAST v14.0 for *Enterobacterales* were applied [23]. *Escherichia coli* ATCC 25922 and *Pseudomonas aeruginosa* ATCC 27853 served as quality control strains. The susceptibility profiles of the isolates were confirmed using the VITEK® 2 automated system for antimicrobial susceptibility testing, employing AST-GN73 cards following the manufacturer’s instructions.

### Whole genome sequencing-based identification

#### Whole genome sequencing, assembly, and annotation

DNA was extracted following the manufacturer’s instructions using QIAamp DNA Kits (Qiagen, Hilden, Germany). WGS and library construction were carried out by BGI Tech Solutions Hong Kong Co., Ltd., China, using DNBseq™ sequencing technology. Quality control and trimming of the raw reads were completed in the pre-assembly stage using BGI’s SOAPnuke software [24]. The filtered reads were de novo assembled using the genome assembly tool provided by the Bacterial and Viral Bioinformatics Resource Center (BV-BRC) [25]. The assemblers Unicycler version 0.4.8 [26] and PlasmidSPAdes were used for the genome and plasmids assembly, respectively. The resulting draft genome was submitted to the NCBI Prokaryotic Genome Annotation Pipeline for gene annotation. Gene sequences were assigned to Kyoto Encyclopedia of Genes and Genomes (KEGG) Orthology (KO) groups using GhostKOALA, which maps genes to KEGG pathways to identify their biological roles [27].

#### 16S rRNA-based identification

The full-length 16S rRNA gene sequence (1,454 bp) was obtained from the WGS of strain DFU52^T^, while only a partial sequence (913 bp) was available for strain DFU6. Both sequences were submitted to the National Center for Biotechnology Information (NCBI) database (www.ncbi.nlm.nih.gov), with accession numbers PQ592516.1 and PQ592533.1, respectively. Pairwise alignment of the two sequences was performed using the Nucleotide Basic Local Alignment Search Tool (BLASTn) [28]. The gene sequences were also analyzed using the 16S rRNA-based identification service on the EzBioCloud server [10] to determine closely related type strains. Further alignment was carried out with entries from the NCBI using the Nucleotide Basic Local Alignment Search Tool (BLASTn). This search targeted the NCBI 16S ribosomal RNA Database for Bacteria and Archaea type strains, a curated resource that includes representative 16S rRNA sequences from bacterial and archaeal type strains [11]. To further ensure comprehensive results, a BLASTn search was also conducted against the NCBI nucleotide collection (nr/nt) database to find the closest species whose genes were not present in the 16S rRNA database.

#### Genome-based taxonomy

To begin the genome-based taxonomy analysis, the Type Strain Genome Server (TYGS) provided by DSMZ (https://tygs.dsmz.de/) was used (accessed on November 10, 2024) to identify the closest related type strain genomes. Using the MASH algorithm [29], a rapid estimation of intergenomic similarity was obtained by comparing the draft genome sequence with all type strains available in the TYGS database. The most similar type strains were selected to construct a phylogenomic tree. For the phylogenomic analysis, pairwise comparisons among the best-matching genomes were performed using the Genome BLAST Distance Phylogeny (GBDP) method [30], with precise intergenomic distances calculated using the ‘trimming’ algorithm and the d5 distance formula [30]. A balanced minimum evolution tree was then built using FASTME 2.1.6.1 with SPR post-processing [31]. Branch support was evaluated through 100 pseudo-bootstrap replicates. The resulting tree was rooted at the midpoint and visualized with PhyD3 [32]. Species and subspecies clustering is done using dDDH cut-off values of 70% and 79%, respectively. In the meantime, the genome-based taxonomy of the strains was investigated by tetra correlation search (TCS) against JSpeciesWS continuously updated reference database, accessed on November 9, 2024 [33]. After confirming that our strains belong to the genus *Providencia*, their taxonomic placement relative to the type strains of all known *Providencia* species was verified by calculating the Overall Genome Relatedness Indices (OGRIs), as previously recommended [34]. The calculated OGRIs included the Average Nucleotide Identity (ANI) using the alignment algorithms MUMmer (ANIm) and BLAST+ (ANIb), along with the correlation index of the Tetra-nucleotide signatures (Tetra). All calculations were performed using JSpeciesWS tools [33]. Additionally, digital DNA–DNA hybridization (dDDH) values were determined using the Genome-to-Genome Distance Calculator (GGDC) 3.0, accessible at https://ggdc.dsmz.de/ggdc.php [35].

### Taxonomic reassessment of the species *P. vermicola*

As of our analysis date (November 10, 2024), only 59 *P. vermicola* genomes were identified in the NCBI genome database, all of which were downloaded for a genome-based taxonomic reassessment (Supplementary Table 1). Our analysis began with constructing a preliminary phylogenetic tree for the *P. vermicola* genomes, including our strains DFU6 and DFU52^T^, using the reference sequence alignment-based phylogeny builder (REALPHY) [36]. This free online tool enables phylogenetic inference from WGS data. All *P. vermicola* genomes were mapped to the reference genome *P. vermicola* DSM17385^T^ (GenBank accession: JAGSPI010000019.1) via Bowtie2, with multiple sequence alignments generated from these mappings. From these alignments, a SNP-based phylogenetic tree was inferred using PhyML, employing *Klebsiella pneumoniae* subsp. *pneumoniae* MGH 78,578 (GenBank accession: NC_009648.1) as an outgroup. The tree was visualized using the interactive tree of life (iTOL) online tool v6.7 (https://itol.embl.de/itol.cgi). Genomes with branch lengths greater than zero (33 genomes) were then uploaded to TYGS (which accepts a maximum of 50 user genomes), along with our strains DFU6 and DFU52^T^, for species and subspecies clustering.

### Multilocus sequence typing (MLST)

The multilocus sequence types (MLST) of the isolates were determined using the PubMLST database for *Providencia* spp. This database employs the MLST scheme outlined by Juneja and Lazzaro [37], which is based on five housekeeping genes: *fusA*, *gyrB*, *ileS*, *lepA*, and *leuS*.

### Resistome, virulome, and mobilome analysis

The web portal Resistance Gene Identifier (RGI) version 6.0.3, hosted by the Comprehensive Antibiotic Resistance Database (CARD) [38], was utilized to identify genes that exhibit at least 80% coverage and 95% identity with known resistance genes listed in the CARD database version 3.3.0. Virulence genes were identified using VFanalyzer, a pipeline for screening known and potential VFs in bacterial genomes. The tool is provided by the Virulence Factors of Pathogenic Bacteria (VFDB) database [39]. The annotated contigs were also examined using SnapGene software version 5.1.3.1 from Insightful Science (http://www.snapgene.com) to explore the genetic environment surrounding various resistance and virulence genes.

Mobile genetic elements were identified using MobileElementFinder tool v1.0.3 [40] hosted by the Center for Genomic Epidemiology. VRprofile web server [23] was also used for the same purpose together with predicting the mobility of the identified plasmid sequences.

The draft genomes were mapped against the genome of *P. vermicola* strain P13 (GenBank accession: CP097327.1) using IslandViewer4 webtool (http://www.pathogenomics.sfu.ca/islandviewer/) [41] to identify the genomic islands. The integrative and conjugative elements (ICEs) were identified using ICEfinder, a web-based tool hosted by the ICEberg database that includes comprehensive information about bacterial ICEs [42]. Prophage regions were identified using PHASTEST (PHAge Search Tool with Enhanced Sequence Translation) webtool [43].

### Phenotypic analysis of DFU52^T^, the type strain of *P. pseudovermicola sp. nov.*

Gram staining was performed to confirm the Gram reaction and examine the morphology of the isolate. The isolate was cultured under aerobic and anaerobic conditions on various media, including MacConkey agar, Luria Bertani (LB) agar, Trypticase Soy agar (TSA), and Müller-Hinton agar (MHA). The growth was evaluated in Trypticase Soy broth across a range of temperatures (4 °C to 45 °C), pH levels (4–10), and salinity concentrations (0–10%). Sporulation ability was assessed using thermal shock at 80 °C for 30 min. Enzymatic activities, such as catalase and oxidase, were tested following previously described methods [44]. The complete biochemical profile of DFU52^T^ was determined using the VITEK® 2 system.

## Results

### Preliminary identification and antimicrobial susceptibility profiles

Both DFU6 and DFU52^T^ were identified as *P. stuartii* using the VITEK® 2 and MALDI-TOF-MS identification system.

Both DFU6 and DFU52^T^ were classified as MDR, exhibiting non-susceptibility to at least one antimicrobial agent in three or more antimicrobial classes, following the MDR phenotype definition provided before [45]. The complete antimicrobial susceptibility profiles of the isolates are presented in Table 1.

**Table 1:** Antimicrobial susceptibility profiles of DFU6 and DFU52^T^

Antimicrobial Class	Antimicrobial agents	Susceptibility (MIC μg ml^− 1^)	
		DFU52^T^	DFU6
Penicillins	Ampicillin	R (≥ 32)	R (≥ 32)
	Ampicillin/sulbactam	R (≥ 32)	R (≥ 32)
	Piperacillin/tazobactam	R (64)	R (64)
Cephalosporins	Cefazolin	R (≥ 64)	R (≥ 64)
	Cefoxitin	R (≥ 64)	R (≥ 64)
	Ceftazidime	R (≥ 64)	R (≥ 64)
	Ceftriaxone	R (16)	R (16)
	Cefepeme	S (2)	R (≥ 64)
Monobactams	Aztreonam	S (2)	S (2)
Carbapenems	Meropenem	R (≥ 16)	S (0.5)
Lipopeptides	Colistin*	R (≥ 256)	R (≥ 256)
Aminoglycosides	Amikacin	R (≥ 64)	R (≥ 64)
	Gentamicin	R (≥ 16)	R (≥ 16)
	Tobramycin	R (≥ 16)	R (≥ 16)
Tetracyclines	Tetracycline	R (ND)	R (ND)
	Tigecycline	R (ND)	R (ND)
Fluoroquinolones	Ciprofloxacin	R (≥ 4)	R (≥ 4)
	Levofloxacin	R (≥ 8)	R (≥ 8)
Folate pathway antagonists	Trimethoprim/ sulfamethoxazole	S (≤ 2/38)	R (4/76)
Phenicols	Chloramphenicol	R (ND)	R (ND)
Fosfomycins	Fosfomycin	R (ND)	R (ND)
Nitrofurans	Nitrofurantoin	R (128)	R (ND)

Colistin MIC was determined by broth microdilution assay; ND, Not determined

### Genome assembly and annotation metrics

The assembly features of the genomes of DFU6 and DFU52^T^ revealed a genome size of 4,416,300 bp and 4,423,558 bp, respectively, with G + C content values of 40.78% for DFU6 and 41.16% for DFU52^T^. DFU6 draft genome contained 50 contigs with an N50 of 735,893 bp and an L50 of 2, while DFU52^T^ had 72 contigs with an N50 of 172,528 bp and an L50 of 9. Regarding genome quality, the completeness was 99.9% for DFU6 and 100% for DFU52^T^, with contamination levels of 0.4% and 0.7%, respectively. Annotation features showed a total of 4,024 genes in DFU6 and 4,084 genes in DFU52^T^, including 3,881 and 3,937 protein-coding CDSs and 79 and 78 RNA genes, respectively. DFU6 and DFU52^T^ both carried type I-F CRISPR-associated protein-coding genes, with 4 and 3 CRISPR arrays, respectively. The functional annotations of the draft genomes are shown in Supplementary Table 2.

### 16S rRNA-based identification

The pairwise alignment of the partial sequence of the 16S rRNA gene of DFU6 showed 100% identity to that of DFU52^T^. Upon uploading the full 16S rRNA sequence of DFU52^T^ to the Ezbiocloud 16S-based ID tool, the highest similarity score (99.66%) was obtained with *P. manganoxydans* LLDRA6. Alignment of the full sequence against the NCBI rRNA type strains/16S ribosomal RNA database revealed the highest match with *P. huaxiensis* strain WCHPr000369 (NR_174258.1) with 98% coverage and 99.08% identity, followed closely by *P. vermicola* strain OP1 (NR_042415.1) with 98% coverage and 99.21% identity.

nBLAST analysis of the full 16S rRNA sequence against the nucleotide (nr/nt) database showed 100% identity and coverage with seven *Providencia* strains, four of which were identified to species level as *P. vermicola*. Other strains identified as *P. vermicola* showed slightly lower identity percentages (99.29–99.87%). This discrepancy in 16S rRNA gene-based identification indicated a need for further analysis using whole-genome data.

### Genome-based phylogeny and taxonomy

**Fig. 1:**
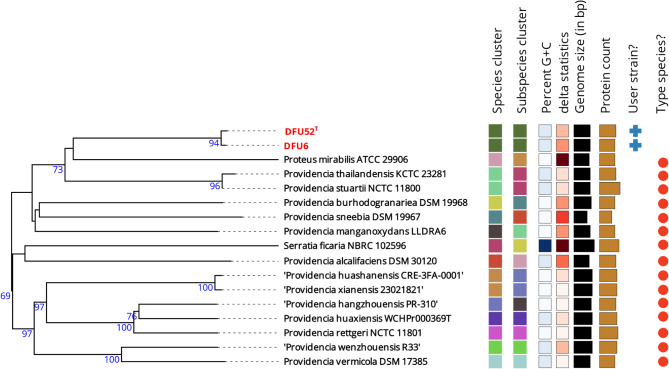
Phylogenomic tree constructed using GBDP distances derived from the genomes of TYGS type strains most closely related to DFU6 and DFU52^**T**^. Clusters representing species and subspecies are defined by dDDH thresholds of 70% and 79%, respectively. Strains from the current study are highlighted in bold red within the tree

The phylogenomic tree presented in Fig. 1, which includes DFU6 and DFU52^T^ alongside type strains from the TYGS database, demonstrates that DFU6 and DFU52^T^ form a distinct cluster at the species and subspecies levels. Notably, they do not cluster with any type strains from other species in the database, including the *P. vermicola* type strain DMS17385^T^.

**Fig. 2:**
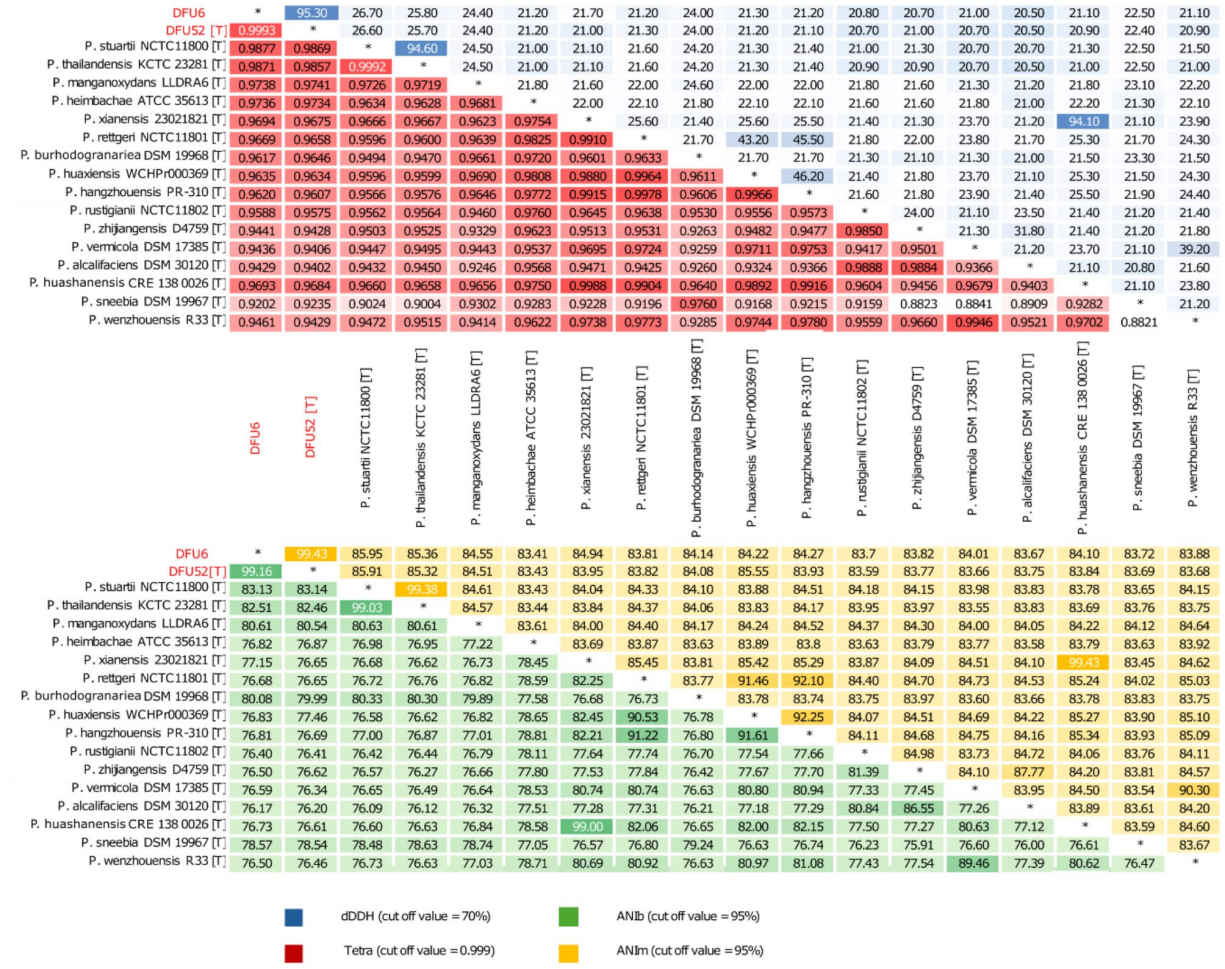
Heatmaps illustrating the results of all-versus-all OGRIs analysis for *Providencia* strains from the current study, alongside type strains of all recognized *Providencia* species

All genomes found in the JSpeciesWS genome database showed a Z-Score of less than 0.999 set by the server as the cut-off value for species assignment. The highest Z-score was found for *P. stuartii* 50655837 with a value of 0.98781. The genomes of the type strains of all currently known *Providencia* species were included in all-versus all OGRIs analysis as shown in Fig. 2. All OGRIs of DFU6 and DFU52^T^ to all type strains including *P. vermicola* type strain DMS17385^T^, were below the species delineation thresholds.

### Taxonomic reassessment of the species *P. vermicola*

The significant discrepancies between the 16S rRNA analysis and genome-based taxonomy, regarding the similarity to *P. vermicola*, prompted additional analyses to confirm the taxonomic positioning of *P. vermicola* genomes available in the NCBI database, whose 16S rRNA genes shared 100% identity with those of our strains.

**Fig. 3:**
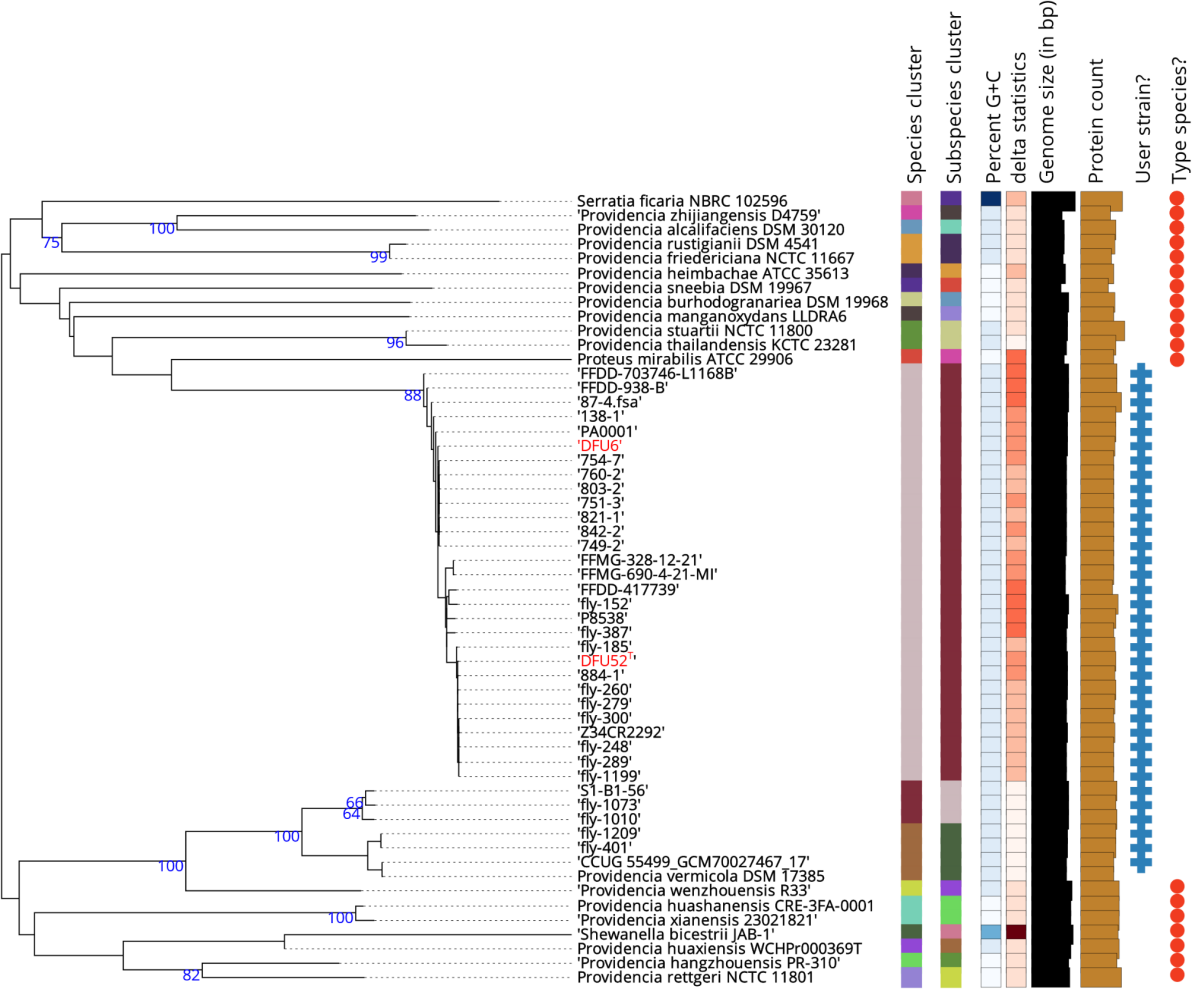
Phylogenomic tree constructed using GBDP distances derived from DFU6, DFU52^T^, and other *P. vermicola* genomes retrieved from the NCBI database alongside most closely related TYGS type strains. Clusters representing species and subspecies are defined by dDDH thresholds of 70% and 79%, respectively. Strains from the current study are highlighted in bold red within the tree

The preliminary SNP-based phylogenetic tree, illustrated in Supplementary Fig. 1, was constructed using our strains in conjunction with all available *P. vermicola* genomes (*n* = 59). This analysis facilitated the selection of 33 *P. vermicola* genomes with branch lengths greater than zero for TYGS species and subspecies clustering, alongside type strains deposited in the TYGS database. As illustrated in Fig. 3, the *P. vermicola* genomes were categorized into three distinct species clusters. Notably, the majority of *P. vermicola* genomes clustered with our strains, which are distinctly separated from the cluster containing the *P. vermicola* type strain DMS 17385^T^. Our analysis confirms that at least 56 strains were misidentified as *P. vermicola* and that their dDDH values compared to *P. vermicola* DSM 17385^T^ are far below the species delineation cut-off values. Based on our analysis, only fly-401 and fly-1209 correctly belong to the species *P. vermicola*. Additionally, the strains fly-1010, fly-1073, and strain S1-B1-56 belong to a novel species that has yet to be identified.

### Multilocus sequence types of DFU6 and DFU52^T^

DFU6 carried novel alleles of *gyrB* (allele 61), *ileS* (allele 73), *lepA* (allele 60), and *leuS* (allele 61), resulting in the unique allelic profile [*fusA9* - *gyrB61* - *ileS73* - *lepA60* - *leuS61*], which was submitted to the MLST database and designated as ST29. Similarly, DFU52^T^ carried the novel alleles *ileS* (allele 74) and *leuS* (allele 60), forming the distinct allelic profile [*fusA9* - *gyrB31* - *ileS74* - *lepA9* - *leuS60*], which was submitted to the MLST database and designated as ST41.

### Resistance genes and associated MGEs

The genomes of DFU6 and DFU52^T^ contained a diverse array of resistance genes, with some predicted to be located on the chromosomes and others associated with MGEs. Genes encoding efflux pumps from all six currently recognized efflux pump classes were identified in both genomes. Additional genes with the potential to confer resistance to various classes of antimicrobial agents are detailed in Table 2.

**Table 2:** Antimicrobial resistance genes carried by DFU6 and DFU52^T^

Antimicrobial class	Antimicrobial Resistance Genes	
	DFU6	DFU52^T^
ß-lactams	*bla*_*CTX−M−14*_ (Class A)	*bla*_CMY−6_ (Class C) - *bla*_NDM−1_ (Class B)
Aminoglycosides	*aadA– armA - aac(2’)-Ia*	*rmtC - aac(6’)-Ib10*, *aac(2’)-Ia - aph(3’)-Ia*
Fluoroquinolones	*qnrD1*	*qnrD1*
Macrolides	*msrE– mphE*	-
Lincosamides	*lnuF*	-
Sulfonamides	*sul1*	*sul1*
diaminopyrimidines	*dfrA1 - dfrA15*	-
Phenicols	*catA*	*catA*
Disinfectants	*qacEdelta1*	*qacEdelta1*

**Fig. 4:**
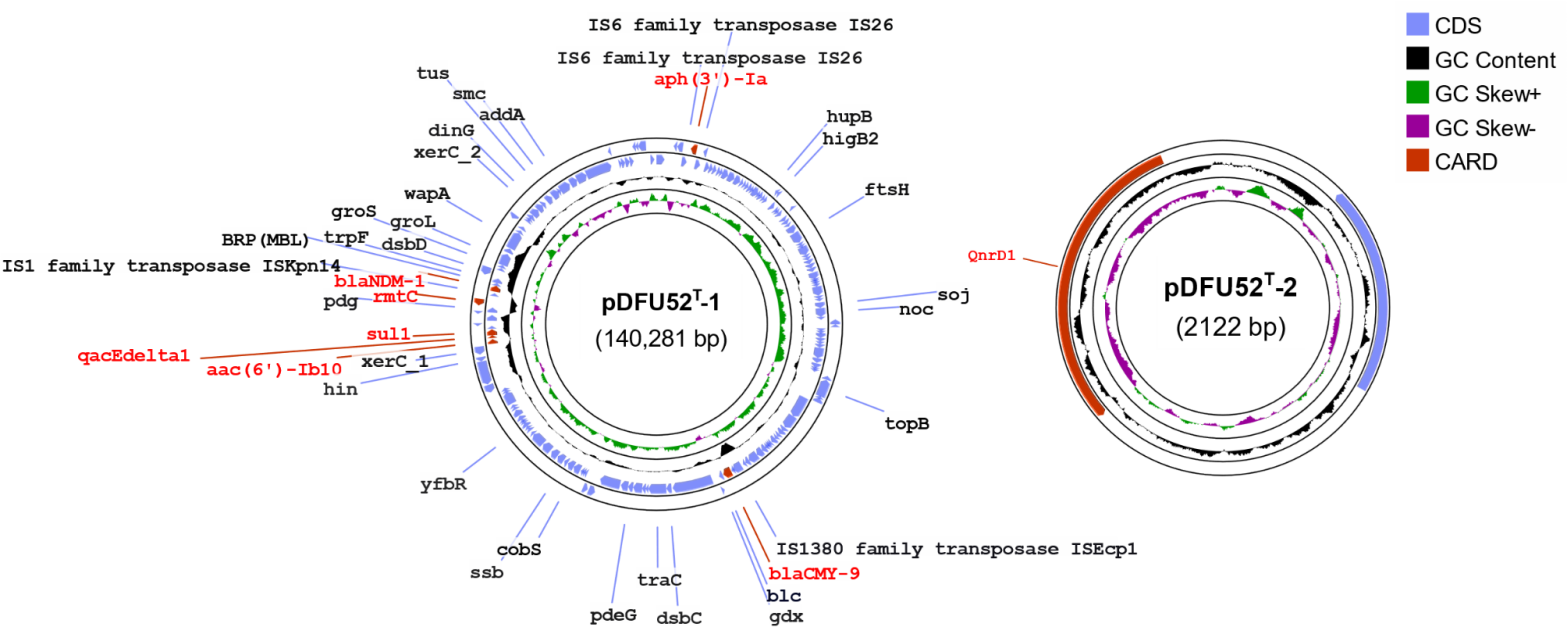
Circular maps illustrating the resistance plasmids carried by DFU52^T^. The unlabelled arrows indicate open reading frames that encode hypothetical proteins. This figure was generated using the Proksee online tool [46]

DFU52^T^ harbored a 140,281 bp IncA/C2 plasmid, designated pDFU52^T^_1, as depicted in Fig. 4. This plasmid was predicted to be conjugative and carried seven resistance genes conferring resistance to β-lactams, including carbapenems, as well as aminoglycosides, sulfonamides, and disinfectants. Many of the resistance genes were associated with MGEs, enabling potential independent mobility. The aminoglycoside resistance gene *aph(3’)-Ia* was flanked by *IS*26 within the composite transposon *Tn*4352. Additionally, the genes *aac(6’)-Ib10*, *qacEdelta1*, and *sul1* were located within a class I integron, while *bla*_CMY−6_ was linked to the insertion sequence *ISEc*p1.

Both DFU6 and DFU52^T^ harboured the *qnrD1* gene on a small, 2,122 bp non-mobilizable plasmid (Fig. 4). This plasmid exhibited high similarity to other small plasmids found in bacterial strains from the genera *Providencia*, *Salmonella*, *Proteus*, *Morganella*, and *Moellerella*.

In DFU6, the resistance genes *aadA*, *dfrA1*, and *lnuF* were associated with a class 2 integron and were carried on a 84,533 contig that showed 99.99% similarity to *P. rettgeri* YPR31 plasmid pYPR31 (GenBank accession: CP053897.1) and *Proteus mirabilis* HBRJC7FQ plasmid pHBRJC7 (GenBank accession: MK630213.1). Moreover, the genes *armA*, *msrE*, and *mphE* were identified on 10,530 bp contig that showed 99.99% similarity to resistance plasmids carried by *Enterobacteriaceae* species. The full sequence of the resistance plasmids carried by DFU6 could not be identified. A genomic island (GI) was identified at the 3’ end of the *mnmE* gene, which encodes a tRNA modification GTPase, in DFU6. A partial sequence of the GI, spanning 21,926 base pairs, was identified (Supplementary Fig. 2). This sequence exhibited the highest similarity to *Proteus mirabilis* genomic island 2 (PGI2) (GenBank accession: MK847916.1), with 99% coverage and 99.46% identity. Only one resistance gene (*dfrA15*) was associated with the class I integron carried by the genomic island.

**Fig. 5:**
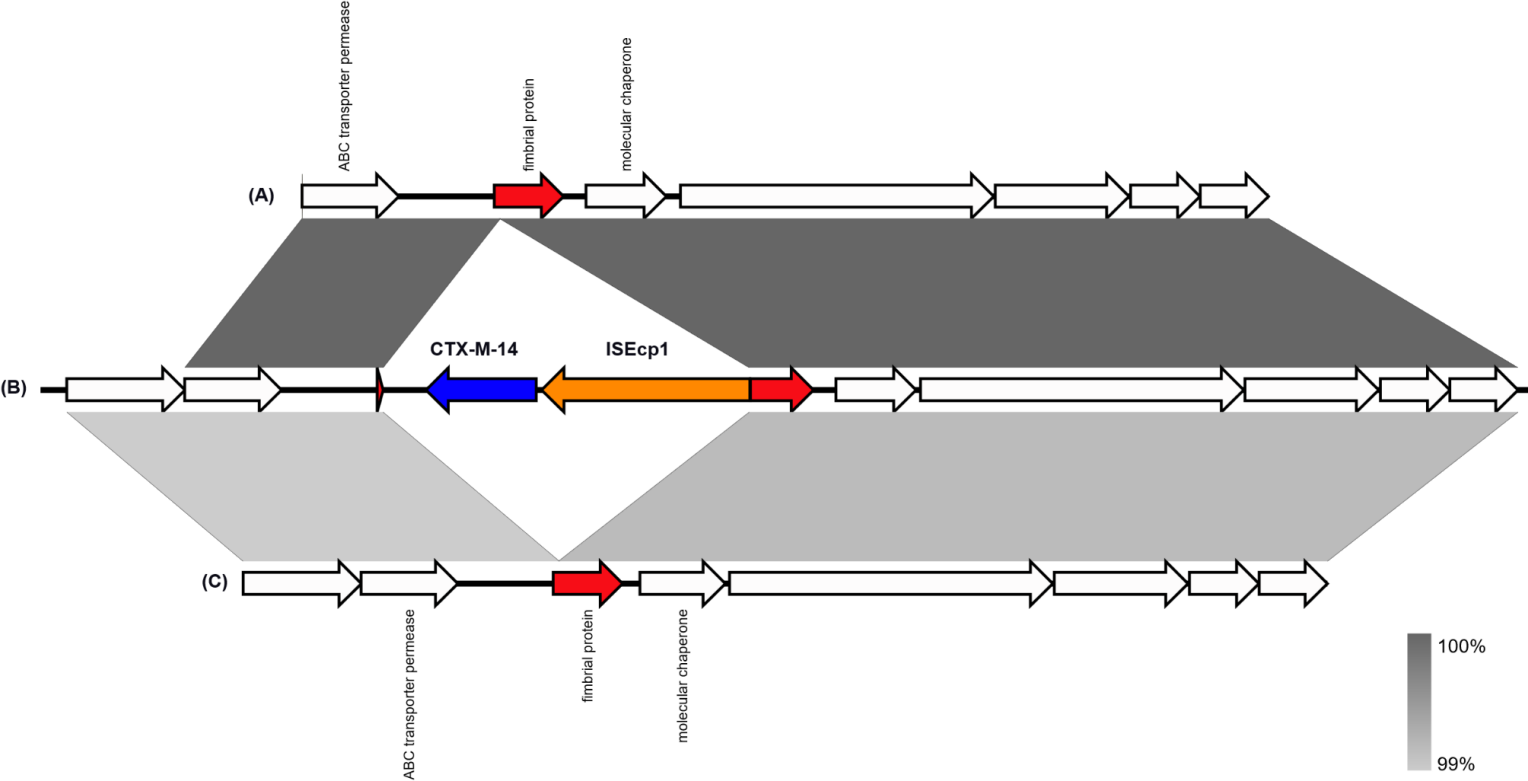
Gene maps depicting the IS*Ecp*1-mediated *bla*_CTX−M−14_ insertion in DFU6 (**B**), compared to *P. stuartii* strain CMC-4104 chromosome (GenBank Accession: CP095443.1 - region: 1234044..1241727) (**A**) and *P. vermicola* P8538 chromosome (GenBank accession: CP048796.1– region: 1167541..1176163) (**C**). Arrows represent open reading frames and are labelled by their predicted protein products. Grey panels between maps correspond to the similarity percent between the chromosomal regions. The figure was created by Easyfig version 2.2.5

Interestingly, DFU6 carried an IS*Ecp*1-mediated insertion of *bla*_CTX−M−14_ with a 5 bp target site duplication (TCCTT), as shown in Fig. 5. The genes were inserted into a fimbrial protein-coding gene with no similarity in the NCBI nucleotide database.

While most resistance genes were predicted to be associated with MGEs, the genes *aac(2’)-Ia*,* ampC*, and *catA* were identified on the chromosomes of DFU6 and DFU52^T^ and showed no association with MGEs.

### Virulence genes and associated MGEs

The functional annotation of the draft genomes revealed that both DFU6 and DFU52^T^ carried at least 74 genes associated with secretion systems and 50 genes encoding bacterial motility proteins. DFU6 encoded 371 transporter-related genes, slightly more than the 364 identified in DFU52^T^. Additionally, DFU6 harbored 29 genes linked to prokaryotic defense systems, compared to 30 in DFU52^T^, as detailed in Supplementary Table 2.

BLAST analysis of the draft genomes against the VFDB was performed to identify genes associated with experimentally confirmed virulence factors. This allowed the identification of virulence factors involved in adherence, motility, secretion systems, metal uptake, immune evasion, and stress survival. The genomes of DFU6 and DFU52^T^ encoded genes for at least five types of fimbriae and pili, including type I fimbriae, type 3 fimbriae, type IV pili, *E. coli* common pilus (ECP), and P fimbriae. Additionally, the genomes harbored genes for five secretion systems: type I, type III and type VI secretion systems, as well as the Sec and Tat secretion pathways. All virulence genes identified in the genomes of DFU6 and DFU52^T^ are listed in Supplementary Table 3.

Additional virulence genes identified from the annotations include those encoding the HlyD family type I secretion (T1SS) periplasmic adaptor subunit, the type I secretion system permease/ATPase, and the type IV secretion system. Both genomes also contained a hemolysin family protein with 59% amino acid sequence identity to the HlyA hemolysin protein, as well as a ShlB/FhaC/HecB family hemolysin secretion/activation protein. Additionally, the quorum sensing QseBC two-component system was encoded by both genomes.

**Fig. 6:**
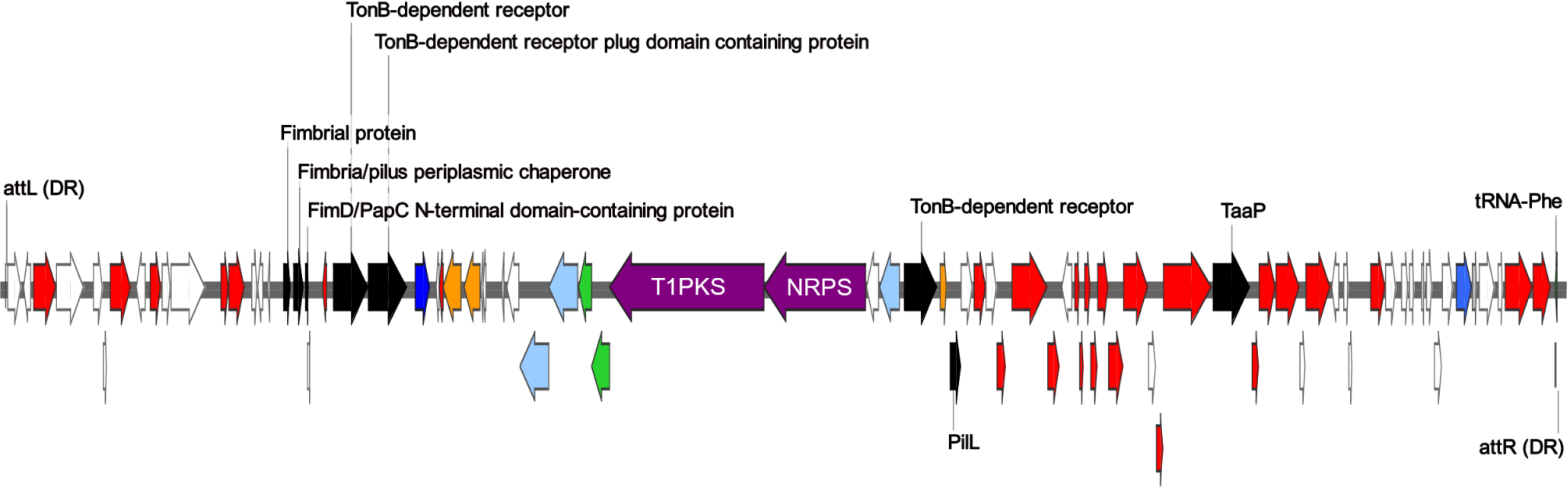
Genetic map illustrating the organization of the PAI carried by DFU6 and DFU52^T^. Red arrows indicate genes involved in the conjugation and integration of the island, black arrows denote virulence genes, orange arrows represent insertion sequences, blue arrows highlight genes encoding ArdC family antirestriction proteins, purple arrows correspond to core biosynthetic genes, green arrows signify additional biosynthetic genes, light blue arrows indicate transport-related genes, and white arrows represent all other genes

An ICE was identified in the draft genomes of DFU6 and DFU52^T^, showing 99.99% identity to PAIs found in *P. stuartii* and other members of the order *Enterobacteriales*, including *Escherichia coli*, *Klebsiella pneumoniae*, *Morganella morganii*, and *Proteus mirabilis*. The island was integrated into the phenylalanine tRNA gene (*pheV*), with total sizes of 92,644 bp in DFU6 and 92,668 bp in DFU52^T^, sharing a sequence identity of 99.92%. As depicted in Fig. 6, the islands contained fimbriae-coding loci with predicted protein products showing 58% identity to F17 fimbriae produced by *Escherichia coli* 111KH86. They also harbored genes encoding a TonB-dependent receptor, the type IV pilus biosynthesis protein PilL, and the trimeric autotransporter adhesin of *Proteus* (TaaP). Additionally, the islands carried biosynthetic gene clusters for type I polyketide synthase (T1PKS) and non-ribosomal peptide synthase (NRPS) production. Genes coding ArdC family antirestriction proteins were also found within the island.

### Prophage regions

**Fig. 7:**
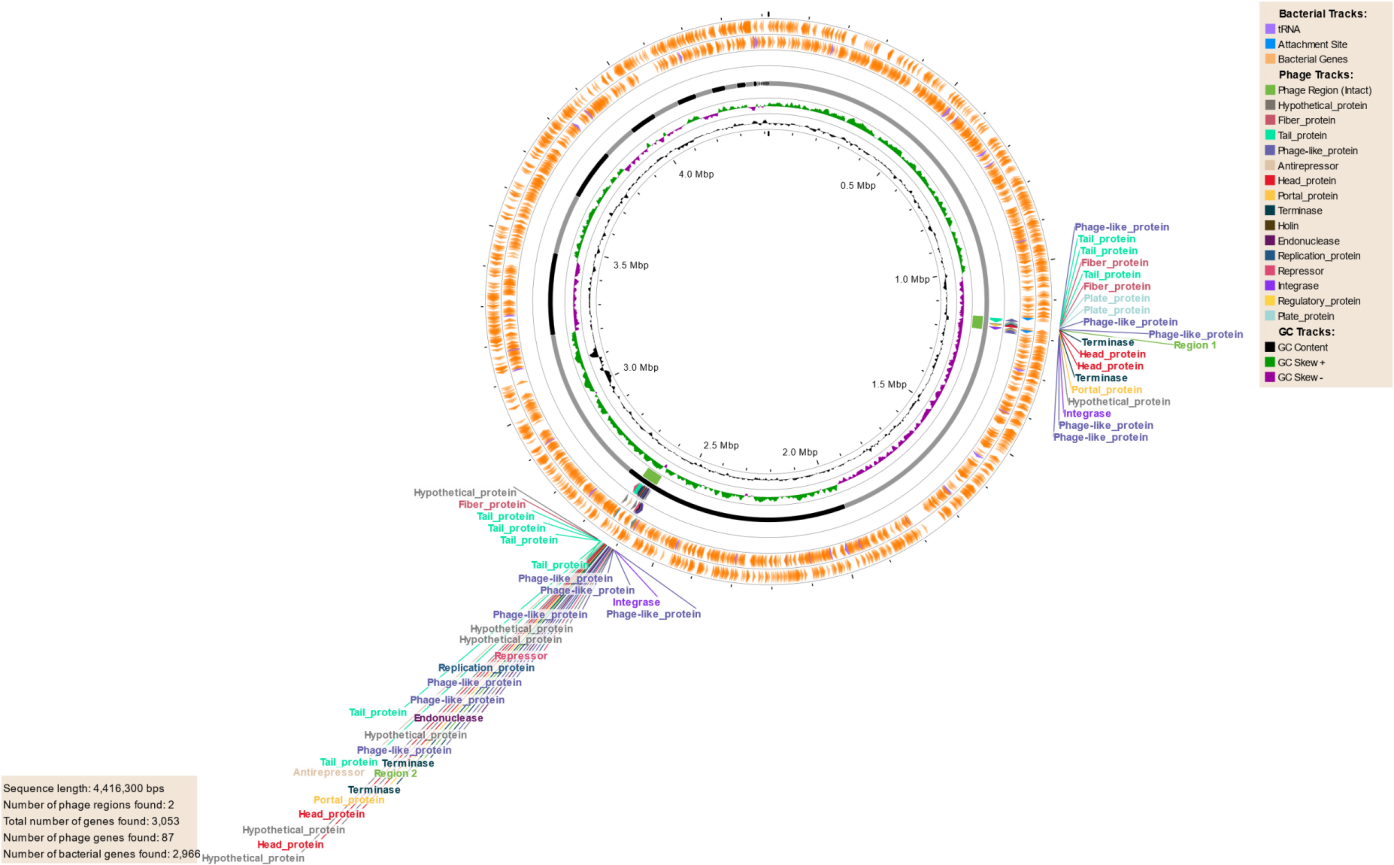
Prophage regions identified in the genome of DFU6. The figure was created by PHASTEST tool

PHASTEST analysis of the DFU6 draft genome identified two intact prophage regions, with genes most commonly matching Entero_phiT5282H (NC_049429) and Cronob_phiES15 (NC_018454) (Fig. 7). In contrast, the DFU52^T^ draft genome contained three questionable prophage regions, with genes most commonly matching Burkho_BcepB1A (NC_005886), Cronob_phiES15 (NC_018454), and Escher_HK75 (NC_016160). A detailed description of the prophage regions identified in DFU6 and DFU52T is provided in Supplementary Table 4.

### Phenotypic features of DFU52^T^, the type strain of *P. pseudovermicola* sp. nov

DFU52^T^ are Gram-negative, non-spore-forming rods that exhibited robust growth under both aerobic and anaerobic conditions on all tested culture media. The isolates demonstrated the ability to grow across the full range of tested temperatures, pH levels, and salinity concentrations. DFU52^T^ tested negative for catalase and oxidase activities. Biochemical features of DFU52^T^ compared to the type strains of other *Providencia* species are shown in Table 3. The full biochemical profile of DFU52^T^ as defined by VITEK® 2 system is shown in Supplementary Table 5.

**Table 3:** Biochemical features of DFU52^T^ and type strains of other *Providencia* species

Biochemical Feature	DFU52^T^	1	2	3	4	5	6	7	8	9	10	11	12	13	14	15	16	17
Indole	−	+	+	+	−	+	+	+	+	+	−	+	+	+	−	−	−	−
Citrate	+	+	−	−	+	+	−	−	−	+	+	−	+	+	+	−	+	+
Urease	+	+	−	−	−	+	+	−	+	−	−	−	+	+	−	+	−	+
ß−galactosidase	−	−	−	−	−	−	−	−	−	−	−	+	−	−	ND	ND	−	−
Ornithine decarboxylase	−	−	−	−	−	−	−	−	−	−	−	+	−	−	ND	ND	−	−
Lysine decarboxylase	−	−	−	−	−	−	−	−	−	−	−	−	−	−	−	ND	−	−
H_2_S production	−	−	−	−	−	−	−	−	−	−	−	−	−	−	−	−	−	−
D-glucose	+	+	+	+	+	+	+	+	+	+	+	+	+	+	+	+	+	+
Saccharose/Sucrose	-	+	−	−	−	−	−	−	−	+	+	−	−	−	ND	−	−	−
D-mannitol	+	+	−	+	−	+	−	−	+	−	+	+	+	+	+	+	+	+
D- sorbitol	-	-	−	−	−	−	−	−	+	−	+	−	−	−	ND	−	−	−
D-cellobiose	-	-	−	−	−	−	−	−	−	−	+	−	ND	ND	ND		−	−
D-Xylose	−	−	−	−	−	−	−	−	−	−	−	+	−	+	ND	−	−	−

1, *P. manganoxydans* LLDRA6^T^; 2, *P. alcalifaciens* DSM 30120^T^; 3, *P. burhodogranariea* DSM 19968^T^; 4, *P. heimbachae* DSM 3591^T^; 5, *P. huaxiensis* KCTC 62577^T^; 6, *P. rettgeri* DSM 4542^T^; 7, *P. rustigianii* DSM 4541^T^; 8, *P. sneebia* DSM 19967^T^; 9, *P. stuartii* DSM 4539^T^; 10, *P. thailandensis* KCTC 23281^T^; 11, *P. vermicola* DSM 17385^T^; 12, *P. hangzhouensis* PR-310^T^; 13, *P. entomophila* IO-23^T^; 14, *P. wenzhouensis* R33^T^; 15, *P. huashanensis* CRE-3FA-0001^T^; *P. zhijiangensis* D4759^T^, *P. xianensis* 23021821^T^. ND, not determined

## Discussion

DFUs are among the most prevalent and severe complications associated with diabetes mellitus, often resulting from peripheral nerve damage and arterial disease [47]. These ulcers frequently become infected, with over half showing signs of infection at the time of presentation, leading to significant morbidity and mortality [48]. The treatment of infected DFUs is challenging due to several factors, including patients’ compromised immune status and poor peripheral circulation. In addition, the polymicrobial nature of the wounds, biofilm formation by infecting pathogens, and the widespread antimicrobial resistance among causative organisms further complicates DFU management [49]. Recent reports from around the world [50–53] including Egypt [54, 55], have documented a rising prevalence of *Providencia* species isolated from DFUs. These infections exacerbate the challenges in managing DFUs due to their intrinsic resistance to several classes of antimicrobial agents [56]. Several key mechanisms mediate intrinsic resistance in *Providencia* species: (a) the inducible expression of AmpC β-lactamases and AAC(2’)-Ia provides resistance to most penicillins, first-generation cephalosporins, and aminoglycosides [57, 58]; (b) tetracycline resistance is driven by the constitutive activity of multidrug efflux pumps [59]; (c) Polymyxin resistance is associated with cell envelope modifications that either inhibit colistin binding to lipid targets or involve lipid A alterations that reduce its binding affinity [60].

In the current study, two MDR *Providencia* sp. isolates were recovered from infected DFUs in diabetic patients from Egypt. A notable discrepancy was observed between phenotypic identification methods and 16S rRNA-based identification. This prompted us to conduct a more in-depth genome-based taxonomic analysis. The 16S rRNA gene analysis revealed 100% identity to *P. vermicola* strains; however, in the GBDP tree generated by TYGS, our strains did not cluster with the type strain of this species. As previously recommended [34, 61], the OGRIs for our strains were calculated in comparison to the type strains of all known *Providencia* species, and all values were well below the established thresholds for species delineation: 95% for ANI, 70% for dDDH, and 0.999 for Tetra [62]. While the reliability of 16S rRNA-based identification has been questioned in several studies [20, 63, 64], the classification error of our isolates was attributed to the misclassification of most *P. vermicola* strains with genomes deposited in the NCBI database, leading to confusion in the taxonomy of this species.

In 2021, Andolfo, et al. [21] published the complete genome of the *P. vermicola* type strain DSM 17385^T^. MLST analysis in the same study using different marker sets revealed that none of the *P. vermicola* genomes published at the time, including the strains P8538, LLDRA6, and G1, truly belonged to this taxonomic species. By the time of our study, the number of genomes in the NCBI database assigned to *P. vermicola* had grown to 59, with 57 genomes submitted after the publication by Andolfo, et al. [21]. Of these, only two strains were correctly classified as *P. vermicola*, while three strains appear to represent a novel species that requires further characterization. The remaining strains, whose genomes clustered with ours, belong to a distinct species, for which we propose the name *P. pseudovermicola*. We attribute this misclassification to the earlier publication of the genome for *Providencia* strain P8538 [65] before that of the *P. vermicola* type strain DSM 17385^T^ [21]. To address this taxonomic confusion, we designate DFU52^T^ (= CCASU-2024-72) as the type strain of the novel species *P. pseudovermicola*, accompanied by its full biochemical characterization and genome analysis.

DFU6 and DFU52^T^ were found to carry acquired resistance genes for multiple classes of antimicrobial agents most of them were associated with mobile genetic elements such as plasmids, integrons, genomic islands, and insertion sequences. Given that *Providencia* species are inherently resistant to many antimicrobial classes, including last-line agents such as colistin and tigecycline [66], the acquisition of additional resistance genes may contribute to the emergence of extensive drug resistance or pandrug resistance. DFU52^T^ harbored a conjugative plasmid containing six resistance genes, including *bla*_CMY6_, *bla*_NDM−1_ and *rmtC*, which confer resistance to cephalosporins, carbapenems, and all members of the aminoglycoside family. Resistance plasmids of *Providencia* species carrying carbapenemase-coding genes have been widely reported [9, 11, 67, 68], underscoring the high capacity of this genus to acquire and disseminate resistance genes. Interestingly, DFU6 carried a unique IS*Ecp*1-mediated insertion of *bla*_CTX−M−14_. IS*Ecp*1 was reported by Poirel, et al. [69] to mobilize the adjacent *bla*_CTX−M_ genes by a transpositional mechanism. This was reported in many Gram-negative species [70, 71] and was confirmed here in DFU6 through the identification of the target site duplication, a hallmark of transposition [72]. Both isolates carried *qnrD* gene on a small non-mobilizable plasmid commonly found in the members of the family *Morganellaceae*, including strains from several *Providencia* species [21, 73–75].

The phenotypic antimicrobial susceptibility profiles of DFU52^T^ and DFU6 aligned with their genotypic characteristics. DFU52^T^ demonstrated susceptibility exclusively to cefepime, aztreonam, and trimethoprim/sulfamethoxazole. Cefepime is a widely recommended treatment for *Enterobacteriales* infections [76]. The susceptibility of the NDM-producing DFU52^T^ to aztreonam can be attributed to the ability of aztreonam to evade Metallobetalactamase-mediated hydrolysis [77]. Its sensitivity to trimethoprim/sulfamethoxazole supports recent efforts to reintroduce older antimicrobial agents into clinical use [78, 79]. Although DFU52^T^ carried the sulfonamide resistance gene *sul1*, no trimethoprim resistance genes were detected. Conversely, DFU6, which harbored *dfrA1* and *dfrA15* trimethoprim resistance genes, was resistant to trimethoprim/sulfamethoxazole. Additionally, the genome of DFU6 did not carry any carbapenemases-coding genes, making it sensitive to meropenem.

While the virulence factors of most *Providencia* species remain largely unexplored, key contributors to the pathogenicity of *Providencia* species that have been studied include cellular adherence, fimbriae production, secretion systems, and urease activity [3, 80]. Most research in this area has focused on *Providencia stuartii*. While most virulence factors have been studied in the context of UTIs [3], the most common *Providencia* infection, some are also relevant to wound infections and DFUs. Genes coding for Type 3 (MR/K) fimbriae, Type I fimbriae, Type 4 pilli, *E. coli* common pilus (ECP), and P fimbriae were carried by the genomes of DFU6 and DFU52^T^. These structures have been previously reported to play crucial roles in host cell adhesion and biofilm formation [81–83]. Biofilm formation is a critical step in the pathophysiology of DFUs. It significantly contributes to disease progression, lesion chronicity, antibiotic resistance emergence, and wound healing challenges [84]. Motility also plays a crucial role in skin infections, enabling movement across the skin surface and facilitating relocation into wounds [85]. At least five motility-related loci were found in the genomes of DFU6 and DFU52^T^. Genes encoding six types of secretion systems were identified, including T1SS, T3SS, T6SS, the Sec secretion pathway, and the Tat secretion pathway. Secretion systems transport substrates including adhesins, enzymes, siderophores, and toxins across bacterial membranes and play an important role in colonizing various eukaryotic hosts [80, 86, 87]. Although the role of the urease enzyme in UTIs caused by *Providencia* species is well-documented [3], its contribution to wound infections remains poorly understood. Urease catalyzes the conversion of urea into ammonia, leading to a significant increase in the pH of the surrounding tissue. This can make wounds more susceptible to opportunistic infections, promoting polymicrobial infections, a common feature of DFUs [88]. Urease also contributes to pathologic effects by causing cytotoxicity to host cells, stimulating inflammatory responses, and enhancing biofilm colonization and persistence [89, 90]. The TCS QseBC quorum-sensing regulatory system, encoded by the genomes of DFU6 and DFU52^T^, is a global regulator of biofilm growth, bacterial motility, and virulence in *E. coli* and *K. pneumoniae* [91].

DFU6 and DFU52^T^ carried a PAI with 99.95% sequence identity to the ICE known as ICE*Pm*1. This PAI is commonly associated with *P. mirabilis*, *P. stuartii*, and *M. morganii* [92]. The self-transmissibility of ICE*Pm*1 has been previously confirmed [93], and the island has shown sequence matches in other species, including *E. coli* and *K. pneumoniae*. It carries genes encoding iron acquisition siderophores and Taap a surface adhesin and autoagglutinin that contribute to the virulence of pathogenic Gram-negative bacteria by facilitating adherence, biofilm formation, invasion, survival within eukaryotic cells, serum resistance, and cytotoxicity [94, 95].

Despite possessing CRISPR systems, both DFU6 and DFU52^T^ harbored prophage genomes. Together with resistance and virulence-associated MGEs, prophages play a crucial role in enhancing the evolutionary potential of the genus *Providencia*.

As we propose DFU52^T^ as the type strain of the novel species *P. pseudovermicola*, we provide a comprehensive biochemical profile to facilitate its identification and differentiation from other *Providencia* species. The biochemical analysis reveals that all *Providencia* species, including DFU52^T^, are negative for H_2_S production and predominantly lack the enzymes ß-galactosidase, ornithine decarboxylase, and lysine decarboxylase, suggesting that these traits may be characteristic of the genus. Glucose fermentation is a consistent metabolic feature across all species, underscoring its essential role in the core metabolic pathways of the genus. However, the ability to ferment other sugars, such as D-mannitol, sucrose, D-xylose, and cellobiose, varies among species. Additionally, variability is observed in traits such as indole production, citrate utilization, and urease activity, which may correspond to species-specific ecological niches or adaptive strategies. These differences provide valuable markers for distinguishing *P. pseudovermicola* DFU52^T^ from other members of the genus.

## Conclusion

This study reports the identification of a novel *Providencia* species, *P. pseudovermicola* sp. nov., isolated from infected DFUs in Egypt. Phylogenomic analyses confirmed *P. pseudovermicola* as a distinct species within the genus *Providencia*, resolving misidentifications of numerous strains previously classified as *P. vermicola*. We propose DFU52^T^ (= CCASU-2024-72) as the type strain for this novel species. The detection of MGEs carrying carbapenem and aminoglycoside resistance genes, coupled with intrinsic resistance to colistin and tigecycline, highlights the significant public health threat posed by the novel species. These findings emphasize the need for enhanced identification and monitoring of *Providencia* species in clinical settings. Moreover, the application of genomic tools to refine species classification and trace the dissemination of antimicrobial resistance genes is essential. Future research should focus on elucidating the pathogenic mechanisms and epidemiological distribution of *P. pseudovermicola*, along with its clinical impact on human infections.

### Description of *Providencia pseudovermicola ***sp. nov.**

*pseudovermicola*. N.L. fem. adj. *pseudovermicola*, referring to the incorrect classification as *Providencia vermicola*, with “*pseudo*-” meaning false or incorrect and “*vermicola*” referring to the original misidentification.

The novel species *P. pseudovermicola* sp. nov. exhibited characteristics typical of the genus *Providencia*, including fermentative metabolism, facultative anaerobic growth, and Gram-negative, rod-shaped cell morphology. It lacked spore formation and tested positive for catalase activity and negative oxidase activity. The strain demonstrated the ability to grow across a wide range of temperatures (4–45 °C), salinity levels (0–10% NaCl), and pH values (4–10). It can grow well on different culture media including MacConkey agar, LB agar, TSA, and MHA.

On TSA, it formed small, round colonies with a smooth, creamy appearance. Acid production was observed from D-glucose and D-mannitol, but not from D-sorbitol, D-cellobiose, D-xylose, or sucrose. The strain tested positive for citrate utilization and urease activity, but negative for ß-galactosidase, ornithine decarboxylase, lysine decarboxylase, indole production, and H_2_S production. These biochemical traits distinguish *P. pseudovermicola* from closely related species within the genus.

The type strain, DFU52^T^ (= CCASU-2024-72), was isolated from an infected DFU in Egypt and has been deposited in the Culture Collection Ain Shams University.

## Electronic supplementary material

Below is the link to the electronic supplementary material.

Supplementary Material 1

## Data Availability

The complete sequence of the 16S rRNA gene from DFU52T has been submitted to the NCBI GenBank database with the accession number PQ592516. In addition, a partial sequence of the same gene associated with DFU6 has been submitted under the accession number PQ592533. Both draft genomes are accessible in the BioProject PRJNA1181229, with the respective accession numbers JBIZWE000000000 for DFU6 and JBIZWF000000000 for DFU52T.

## Abbreviations

ANI: Average nucleotide identity COGs: Clusters of orthologous group
ANIb: Average nucleotide identity calculated by the alignment algorithms BLAST +
ANIm: Average nucleotide identity calculated by MUMmer
dDDH: The digital DNA–DNA hybridization
DFU: Diabetic foot ulcer
GBDP: Genome BLAST distance phylogeny
GI: Genomic island
ICE: Integrative and conjugative element
MDR: Multidrug-resistant
MLST: Multilocus sequence typing
OGRIs: Overall genome-relatedness indices
PAI: Pathogenicity island
Tetra: Tetra-nucleotide signatures
TYGS: Type strain genome server
WGS: Whole genome sequencing
