# Supplementary material for: Providencia pseudovermicola sp. nov.: redefining Providencia vermicola and unveiling multidrug-resistant strains from diabetic foot ulcers in Egypt
Source: BMC Microbiol. 2025 Apr 23;25:238. doi: 10.1186/s12866-025-03927-3 (PMC12016157; doi:10.1186/s12866-025-03927-3)
Supplement: Supplementary file 1 — Supplementary Material 1 [file 12866_2025_3927_MOESM1_ESM.docx]

***Providencia pseudovermicola* sp. nov.: Redefining *Providencia vermicola* and Unveiling Multidrug-Resistant Strains from Diabetic Foot Ulcers in Egypt**

**Supplementary Table 1: Metadata of *P. vermicola* genomes included in the current study**

| **Assembly Accession** | **Organism name** | **Strain Name** | **Release Date** | **Project Accession** |
| --- | --- | --- | --- | --- |
| GCA_010748935.1 | *Providencia vermicola* | P8538 | 2/19/2020 | NA |
| GCA_020381325.1 | *Providencia vermicola* | DSM 17385[T]* | 10/8/2021 | JAGSPI01 |
| GCA_023704235.1 | *Providencia vermicola* | P13 | 6/13/2022 | NA |
| GCA_024655165.1 | *Providencia vermicola* | PA0001 | 8/14/2022 | JANKLM01 |
| GCA_029085295.1 | *Providencia vermicola* | PVA3 | 3/9/2023 | JAPZEK01 |
| GCA_029085305.1 | *Providencia vermicola* | PVA4 | 3/9/2023 | JAPZEJ01 |
| GCA_029085345.1 | *Providencia vermicola* | PVA2 | 3/9/2023 | JAPZEL01 |
| GCA_029085385.1 | *Providencia vermicola* | PVA1 | 3/9/2023 | JAPZEM01 |
| GCA_029542345.1 | *Providencia vermicola* | PVA41-chromosome | 4/3/2023 | NA |
| GCA_036862615.1 | *Providencia vermicola* | Z35CR2240 | 2/23/2024 | JAZHYJ01 |
| GCA_036898205.1 | *Providencia vermicola* | Z34CR2292 | 2/28/2024 | NA |
| GCA_040136595.1 | *Providencia vermicola* | fly-1210 | 6/11/2024 | JBEEQK01 |
| GCA_040136975.1 | *Providencia vermicola* | fly-1187 | 6/11/2024 | JBEERC01 |
| GCA_040136605.1 | *Providencia vermicola* | fly-1211 | 6/11/2024 | JBEEQJ01 |
| GCA_040136865.1 | *Providencia vermicola* | fly-1193 | 6/11/2024 | JBEEQX01 |
| GCA_040139225.1 | *Providencia vermicola* | fly-1073 | 6/11/2024 | JBEEUF01 |
| GCA_040136745.1 | *Providencia vermicola* | fly-1199 | 6/11/2024 | JBEEQS01 |
| GCA_040140285.1 | *Providencia vermicola* | fly-1010 | 6/11/2024 | JBEEWG01 |
| GCA_040136585.1 | *Providencia vermicola* | fly-1209 | 6/11/2024 | JBEEQL01 |
| GCA_964209035.1 | *Providencia vermicola* | FFDD-417739 | 7/30/2024 | CAXOHT01 |
| GCA_964211635.1 | *Providencia vermicola* | FFZB-192B | 7/30/2024 | CAXORS01 |
| GCA_964211645.1 | *Providencia vermicola* | FFZB-192A-Q19 | 7/30/2024 | CAXORQ01 |
| GCA_964210245.1 | *Providencia vermicola* | FFMG-690-4-21-MI | 7/30/2024 | CAXOLV01 |
| GCA_964210885.1 | *Providencia vermicola* | FFDD-938-B | 7/30/2024 | CAXOPF01 |
| GCA_964211395.1 | *Providencia vermicola* | FFMG-328-12-21 | 7/30/2024 | CAXOQF01 |
| GCA_964211745.1 | *Providencia vermicola* | FFZB-160C | 7/30/2024 | CAXOSD01 |
| GCA_964210345.1 | *Providencia vermicola* | FFDD-703746-L1168B | 7/30/2024 | CAXONC01 |
| GCA_964210465.1 | *Providencia vermicola* | FFDD-703746-L1168A | 7/30/2024 | CAXONU01 |
| GCA_041022555.1 | *Providencia vermicola* | 803-2 | 8/3/2024 | JBFTCW01 |
| GCA_041023445.1 | *Providencia vermicola* | 760-2 | 8/3/2024 | JBFTCP01 |
| GCA_041023235.1 | *Providencia vermicola* | 821-1 | 8/3/2024 | JBFTDB01 |
| GCA_041023335.1 | *Providencia vermicola* | 754-7 | 8/3/2024 | JBFTCM01 |
| GCA_041022185.1 | *Providencia vermicola* | 884-1 | 8/3/2024 | JBFTDP01 |
| GCA_041022735.1 | *Providencia vermicola* | 749-2 | 8/3/2024 | JBFTCH01 |
| GCA_041022635.1 | *Providencia vermicola* | 751-3 | 8/3/2024 | JBFTCK01 |
| GCA_041022295.1 | *Providencia vermicola* | 842-2 | 8/3/2024 | JBFTDK01 |
| GCA_041025355.1 | *Providencia vermicola* | 138-1 | 8/3/2024 | JBFSYJ01 |
| GCA_041022315.1 | *Providencia vermicola* | 87-4 | 8/3/2024 | JBFTDN01 |
| GCA_041099255.1 | *Providencia vermicola* | fly-387 | 8/7/2024 | JBFZSM01 |
| GCA_041098495.1 | *Providencia vermicola* | fly-428 | 8/7/2024 | JBFZTX01 |
| GCA_041098615.1 | *Providencia vermicola* | fly-420 | 8/7/2024 | JBFZTR01 |
| GCA_041098715.1 | *Providencia vermicola* | fly-416 | 8/7/2024 | JBFZTN01 |
| GCA_041098775.1 | *Providencia vermicola* | fly-412 | 8/7/2024 | JBFZTJ01 |
| GCA_041098535.1 | *Providencia vermicola* | fly-429 | 8/7/2024 | JBFZTY01 |
| GCA_041090615.1 | *Providencia vermicola* | fly-279 | 8/7/2024 | JBFZOU01 |
| GCA_041090195.1 | *Providencia vermicola* | fly-300 | 8/7/2024 | JBFZPO01 |
| GCA_041090235.1 | *Providencia vermicola* | fly-299 | 8/7/2024 | JBFZPN01 |
| GCA_041090855.1 | *Providencia vermicola* | fly-260 | 8/7/2024 | JBFZOH01 |
| GCA_041098855.1 | *Providencia vermicola* | fly-409 | 8/7/2024 | JBFZTH01 |
| GCA_041090595.1 | *Providencia vermicola* | fly-278 | 8/7/2024 | JBFZOT01 |
| GCA_041091055.1 | *Providencia vermicola* | fly-248 | 8/7/2024 | JBFZNX01 |
| GCA_041093995.1 | *Providencia vermicola* | fly-75 | 8/7/2024 | JBFZIG01 |
| GCA_041098625.1 | *Providencia vermicola* | fly-422 | 8/7/2024 | JBFZTT01 |
| GCA_041090395.1 | *Providencia vermicola* | fly-289 | 8/7/2024 | JBFZPD01 |
| GCA_041098995.1 | *Providencia vermicola* | fly-401 | 8/7/2024 | JBFZSZ01 |
| GCA_041092655.1 | *Providencia vermicola* | fly-152 | 8/7/2024 | JBFZKT01 |
| GCA_041091955.1 | *Providencia vermicola* | fly-194 | 8/7/2024 | JBFZME01 |
| GCA_041092115.1 | *Providencia vermicola* | fly-185 | 8/7/2024 | JBFZLW01 |
| GCA_041359575.1 | *Providencia vermicola* | S1-B1-56 | 8/19/2024 | JBGJLY01 |
| GCA_042678565.1 | *Providencia vermicola* | CCUG 55499* | 10/6/2024 | JBHTKQ01 |

* CCUG 55499 and DMS 17385 are the same strain.

**Supplementary Table 2: Functional classification of genes in DFU6 and DFU52^T^ based on Kegg Orthology analysis**

| **Protein Family** | **KO groups** | **DFU6** | **DFU52^T^** |
| --- | --- | --- | --- |
| **Metabolism** | ko01000 Enzymes | 1013 | 1012 |
|  | ko01001 Protein kinases | 21 | 20 |
|  | ko01009 Protein phosphatases and associated proteins | 4 | 5 |
|  | ko01002 Peptidases and inhibitors | 63 | 61 |
|  | ko01003 Glycosyltransferases | 9 | 9 |
|  | ko01005 Lipopolysaccharide biosynthesis proteins | 31 | 31 |
|  | ko01011 Peptidoglycan biosynthesis and degradation proteins | 38 | 38 |
|  | ko01004 Lipid biosynthesis proteins | 20 | 20 |
|  | ko01008 Polyketide biosynthesis proteins | 5 | 5 |
|  | ko01006 Prenyltransferases | 7 | 7 |
|  | ko01007 Amino acid related enzymes | 40 | 39 |
|  | ko00194 Photosynthesis proteins | 9 | 9 |
| **Genetic information processing** | ko03000 Transcription factors | 89 | 89 |
|  | ko03021 Transcription machinery | 24 | 24 |
|  | ko03019 Messenger RNA biogenesis | 25 | 25 |
|  | ko03011 Ribosome | 54 | 54 |
|  | ko03009 Ribosome biogenesis | 66 | 66 |
|  | ko03016 Transfer RNA biogenesis | 93 | 94 |
|  | ko03012 Translation factors | 21 | 21 |
|  | ko03110 Chaperones and folding catalysts | 43 | 43 |
|  | ko04131 Membrane trafficking | 6 | 7 |
|  | ko04121 Ubiquitin system | 1 | 1 |
|  | ko03051 Proteasome | 1 | 1 |
|  | ko03032 DNA replication proteins | 32 | 32 |
|  | ko03036 Chromosome and associated proteins | 63 | 64 |
|  | ko03400 DNA repair and recombination proteins | 88 | 86 |
|  | ko03029 Mitochondrial biogenesis | 24 | 25 |
| **Signaling and cellular processes** | ko02000 Transporters | 371 | 364 |
|  | ko02044 Secretion system | 74 | 74 |
|  | ko02042 Bacterial toxins | 1 | 1 |
|  | ko02022 Two-component system | 41 | 39 |
|  | ko02035 Bacterial motility proteins | 50 | 50 |
|  | ko04812 Cytoskeleton proteins | 8 | 8 |
|  | ko04147 Exosome | 36 | 37 |
|  | ko02048 Prokaryotic defense system | 29 | 30 |
|  | ko01504 Antimicrobial resistance genes | 17 | 16 |
|  | ko00536 Glycosaminoglycan binding proteins | 0 | 1 |
|  | ko00537 Glycosylphosphatidylinositol -anchored proteins | 1 | 1 |
| **Viral protein families** | ko03200 Viral proteins | 1 | 1 |

**
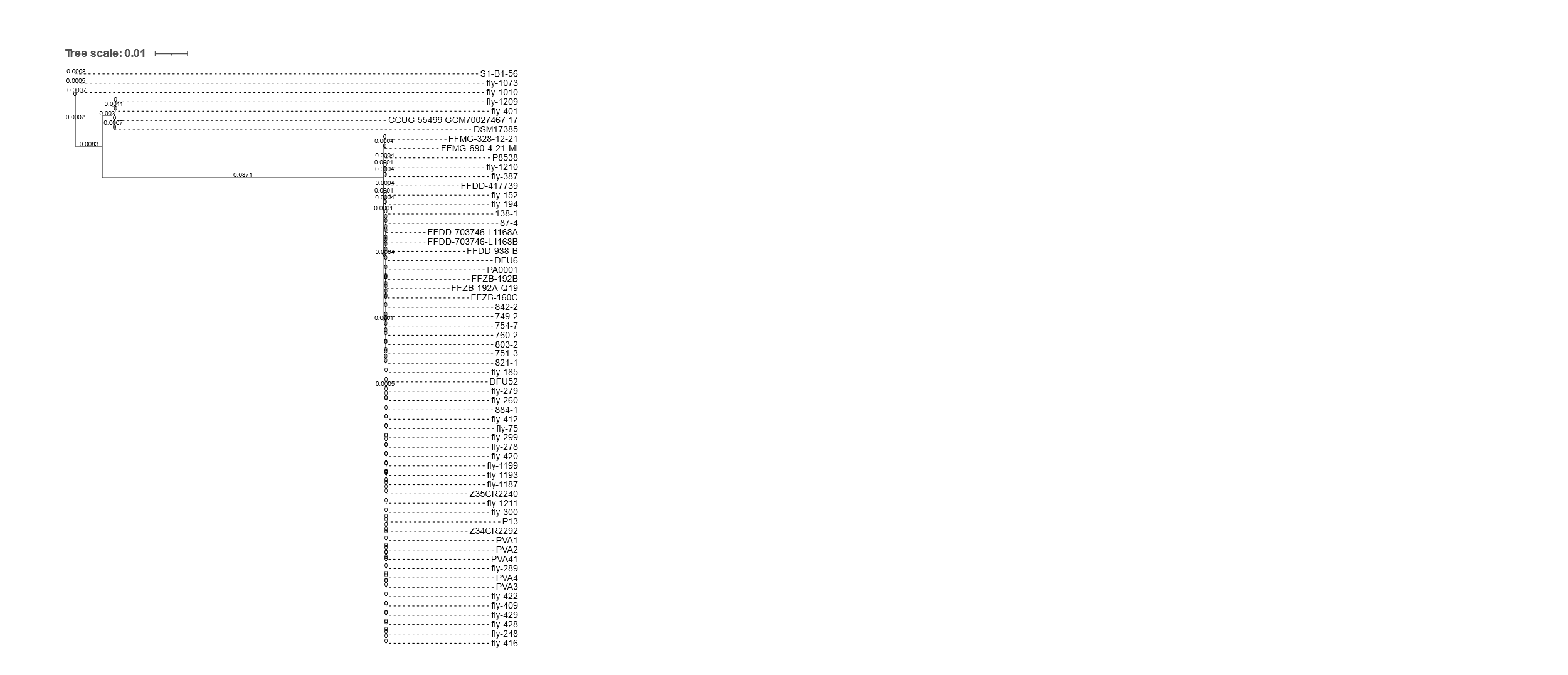
**

**Supplementary Figure 1: SNP-based phylogenetic tree constructed using DFU6 and DFU52^T^, along with other *P. vermicola* genomes available in the NCBI database at the time of analysis.**


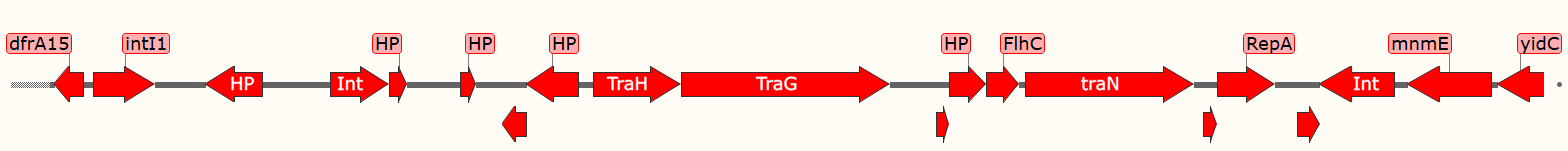


**Supplementary Figure 2: DFU6 GI partial sequence 21,926 bp (Contig 8).** Arrows represent open reading frames. *intI1*, class I integron integrase-coding gene; *dfrA15*, dihydrofolate reductase-coding gene.

**Supplementary Table 3: Predicted virulence genes and associated virulence factors identified in the genomes of DFU6 and DFU52^T^**

| **VF Class** | **Virulence factors** | **Related genes** | **DFU6** | **DFU52^T^** |
| --- | --- | --- | --- | --- |
|  |  |  |  |  |
| **Adherence** | **Type 3 (MR/K) fimbriae** | *mrkABC* | **+** | **+** |
|  | **Type I fimbriae** | *fimABCDEH* | **+** | **+** |
|  | **Type 4 pilli** | *pilABCMNQ* | **+** | **+** |
|  | ***E. coli* common pilus (ECP)** | *ecpABCDE* | **+** | **+** |
|  | **P fimbriae** | *papACD* | **+** | **+** |
| **Motility** | **Flagella** | *flgBCDEFGHIJKLMN* | **+** | **+** |
|  |  | *flhABCD* | **+** | **+** |
|  |  | *fliACDEFGHIJKLMNOPQRSTZ* | **+** | **+** |
|  |  | *motAB* | **+** | **+** |
|  |  | *cheABDMWRYZ* | **+** | **+** |
| **Secretion Systems** | **T3SS** | *sctCEIJNSTW* | **+** | **+** |
|  |  | *escFUVR/yscFUVR/hrpA/hrcUVR* | **+** | **+** |
|  |  | *sicA* | **+** | **+** |
|  |  | *exsA* | **+** | **+** |
|  |  | *fliP* | **+** | **+** |
|  | **T6SS** | *tssABCDEFGHIJKL* | **+** | **+** |
|  |  | *tle1* | **+** | **+** |
|  | **Sec Secretion pathway** | *secABDEFGY* | **+** | **+** |
|  | **Tat Secretion pathway** | *tatABC* | **+** | **+** |
| **Toxin** | **RTX toxin** | *rtxBD* | **+** | **+** |
|  |  | *rtxE* | **+** | **+** |
| **Immune modulation** | **LOS** | *wecA* | **+** | **+** |
|  |  | *hldE* | **+** | **+** |
|  | **LPS** | *Rfb locus* | **+** | **+** |
| **Iron uptake** | **Aerobactin** | *iutA* | **+** | **+** |
|  | **Enterobactin** | *entABCE* | **+** | **+** |
|  | **Yersiniabactin** | *irp12* | **+** | **+** |
|  |  | *ybtPQU* | **+** | **+** |
|  | **Heme transport** | *shuV* | **+** | **+** |
|  | **Heme uptake** | *chuASU* | **+** | **+** |
|  | **Iron-regulated element** | *ireA* | **+** | **+** |
|  | **Iron/manganese transport** | *sitABCD* | **+** | **+** |
|  | **Pyochelin receptor** | *fptA* | **+** | **+** |
| **Magnesium uptake** | **Mg2+ transport** | *mgtAE* | **+** | **+** |
| **Immune evasion** | **Capsule** | *wecA* | **+** | **+** |
|  |  | *wcbN* | **+** | **+** |
|  | **Exopolysaccharide** | *galE* | **+** | **+** |
| **Stress survival** | **Super oxide dismutase** | *sodABC* | **+** | **+** |
|  | **Urease** | *ureABCDEFG* | **+** | **+** |
|  | **Catalase** | *KatA* | **+** | **+** |

**Supplementary Table 4: Prophage regions identified in the draft genomes of DFU6 and DFU52^T^**

| **Region** | **Completeness** | **Score** | **Region Length (Kb)** | **No. of proteins** | **Region position** | **Most common phage (# hit genes count)** | **GC content** |
| --- | --- | --- | --- | --- | --- | --- | --- |
| **DFU6_1** | **Intact** | **150** | **42** | **42** | **Assembly contig 1 (1151641-1193669)** | **PHAGE_Entero_phiT5282H_NC_049429 (14)** | **42.24%** |
| **DFU6_2** | **Intact** | **150** | **55.1** | **87** | **Assembly contig 2 (635605-690718)** | **PHAGE_Cronob_phiES15_NC_018454 (11)** | **41.35%** |
| **DFU52^T^_1** | **Questionable** | **90** | **30.8** | **37** | **Assembly contig 2 (55431-86305)** | **PHAGE_Burkho_BcepB1A_NC_005886 (7)** | **41.55%** |
| **DFU52^T^_2** | **Questionable** | **90** | **11.9** | **17** | **Assembly contig 31 (2-11967)** | **PHAGE_Cronob_phiES15_NC_018454 (8)** | **42.80%** |
| **DFU52^T^_3** | **Questionable** | **70** | **8.2** | **8** | **Assembly contig 33 (393-8547)** | **PHAGE_Escher_HK75_NC_016160 (2)** | **43.14%** |

**Supplementary Table 5: Biochemical profile of DFU52^T^, as defined by VITEK®2 identification system**

| **Test** | **Result** | **Test** | **Result** | **Test** | **Result** |
| --- | --- | --- | --- | --- | --- |
| Ala-phe-pro-arylamidase | - | ß-glucosidase | - | D-tagatose | - |
| Glu-Gly-Arg- arylamidase | - | alpha-glucosidase | - | D-trehalose | + |
| L-pyrrolydonyl-arylamidase | - | ß-N-acetyl-galatosaminidase | + | L-arabitol | - |
| ß-galactosidase | - | alpha-galactosidase | - | D-cellobiose | - |
| ß-N-acetyl-glucoseaminidase | + | Phosphatase | + | D-glucose | + |
| Glutamyl arylamidase pNA | - | ß-glucoronidase | - | D-maltose | - |
| Gamma- glutamyl- transferase | + | Succinate alkalinisation | + | D-mannitol | + |
| ß-xylosidase | - | L-lactate alkalinisation | - | D-mannose | + |
| ß-alanine arylamidase pNA | - | Decarboxylase base | - | D- sorbitol | - |
| L-proline arylamidase | - | L-histidine assimilation | - | Palantinose | - |
| Lipase | - | Coumarate | + | Saccharose/Sucrose | - |
| Glycine arylamidase | - | H_2_S production | - | Fermentation/glucose | + |
| Ornithine decarboxylase | - | O/129 resistance (comp. *vibrio.*) | + | Ellman | + |
| Lysine decarboxylase | - | L-malate assimilation | - | Adonitol | - |
| Tyrosine arylamidase | + | L-lactate assimilation | - | Malonate | - |
| Urease | + | 5-keto-D-gluconate | - | Citrate (sodium) | + |
